# Complete Genome Sequence of High Current-Producing Geobacter sulfurreducens Strain YM35, Isolated from River Sediment in Japan

**DOI:** 10.1128/MRA.00539-21

**Published:** 2021-08-19

**Authors:** Takashi Fujikawa, Yoshitoshi Ogura, Tetsuya Hayashi, Kengo Inoue

**Affiliations:** a Interdisciplinary Graduate School of Agriculture and Engineering, University of Miyazaki, Miyazaki, Japan; b Division of Microbiology, Department of Infectious Medicine, Kurume University School of Medicine, Kurume, Japan; c Department of Bacteriology, Faculty of Medical Sciences, Kyushu University, Fukuoka, Japan; d Department of Biochemistry and Applied Biosciences, Faculty of Agriculture, University of Miyazaki, Miyazaki, Japan; University of Southern California

## Abstract

Here, we report the complete genome sequence of Geobacter sulfurreducens strain YM35, which was isolated from biofilms formed on an anode in a bioelectrochemical system where river sediment was used as an inoculum. The chromosome is 3,745,223 bp with a G+C content of 60.9%. The chromosome contains 3,324 protein-coding genes.

## ANNOUNCEMENT

Geobacter sulfurreducens is one of the high current-producing bacteria, which transfer electrons to extracellular electrodes in their respiration processes (1, 2). The mechanism of the extracellular electron transfer to electrodes in G. sulfurreducens has been extensively studied in the strain PCA, which is the type strain of G. sulfurreducens (3). G. sulfurreducens strains KN400 and YM18, which produce a higher current density than strain PCA, were also isolated, and their complete genome sequences have been determined (4–6). Here, we determined the complete genome sequence of G. sulfurreducens strain YM35, which is another high current-producing strain, isolated from biofilms formed on an anode poised at −0.2 V (versus standard electrode system [SHE]) in a bioelectrochemical system where river sediment was used as an inoculum (T. Fujikawa, Y. Ogura, K. Ishigami, Y. Kawano, M. Nagamine, T. Hayashi, and K. Inoue, submitted for publication).

A frozen stock of strain YM35 (Fujikawa et al., submitted) was inoculated into NBAF medium with acetate (10 mM) and fumarate (40 mM) as the sole electron donor and acceptor, respectively, and cultivated at 30°C, as described previously (7). The medium was bubbled with N_2_-CO_2_ mixed gas (80:20, vol/vol) and autoclaved at 121°C for 30 min. The sterilized anaerobic l-cysteine solution was added to the medium before use (final concentration, 1 mM). Genomic DNA was purified with the genomic-tip 100/G column and genomic DNA buffer set (Qiagen) according to the manufacturer’s instructions. A paired-end library was constructed using the Nextera XT DNA library preparation kit (Illumina). The library was sequenced using an Illumina MiSeq instrument with the MiSeq reagent kit version 2 (Illumina) to obtain 251-bp paired-end reads. An 8-kb mate-pair library was also prepared using the Nextera mate-pair sample preparation kit (Illumina) and sequenced using the MiSeq platform. Low-quality sequences and adapters were removed using Platanus_trim version 1.0.7 (http://platanus.bio.titech.ac.jp/pltanus_trim). Default parameters were used for all software unless otherwise specified. A total of 3,206,380 paired-end and 1,057,520 mate-pair reads were assembled by Platanus version 1.2.1 (8), yielding 1 scaffold containing 33 gaps. The gap-spanning regions were amplified by PCR using the genomic DNA extracted from strain YM35 cells cultured in NBAF medium at 30°C as the template and the amplification primers listed in Table 1. Amplicons were sequenced by Sanger sequencing using the sequencing primers listed in Table 1, and gap sequences (69,183 bp in total) were determined. The coverage of the whole-genome sequence was 137×. The complete genome of strain YM35 consists of a 3,745,223-bp circular chromosome with a G+C content of 60.9%. The genome was smaller than that of strain PCA (3,814,128 bp) and larger than those of strains KN400 (3,726,411 bp) and YM18 (3,714,272 bp). No plasmid was found. Annotation by NCBI Prokaryotic Genome Annotation Pipeline (PGAP) version 5.2 (https://www.ncbi.nlm.nih.gov/genome/annotation_prok) (9) identified 2 rRNA operons, 48 tRNA genes, and 3,324 protein-coding sequences (CDSs). The 3,324 CDSs included 102 genes for *c*-type cytochromes, which was comparable to those of strains PCA, KN400, and YM18.

**TABLE 1:** Primers used for PCR and sequencing of the gaps in the assembled draft genome sequence of strain YM35

Gap no.	Forward primer (5′ to 3′)^*a*^	Reverse primer (5′ to 3′)^*a*^	For use in:	Annealing temp (°C)^*b*^	Extension time (s)^*b*^	In the final closed chromosome		
						No. of Ns	Start position	End position
1	**GGACGAACTACCGGTTTCTTTC**	**CATCCAGGCTGATGTAGACCAT**	PCR amplification, sequencing	60	30	287	225930	226216
2	**CCGGTATACGAGACAACTCCAT**	**CTGGCAGAGAATGATCAAGATG**	PCR amplification, sequencing	60	30	5,772	489062	494833
	CATGTCCGTTGCCGGATCGAAG	AGCAGTGCGGCCGTAGTGAAAC	Sequencing					
	TGTGGTCACAACGTGATCGC	TCGAGACGGTGACGAACTTC	Sequencing					
	TCGAAGATGTGCGGCAGATC	ACAAGGGGTCGTCGGATATC	Sequencing					
	CAACTCCGAATACCACAAAC	GCTGGACCTTTTCAAGGGTC	Sequencing					
		TTCCGCATGAAGTACTCGAG	Sequencing					
3	**CTGAAACGGTACACGGAGGT**	**GATGTCGCTCATGTGGATGC**	PCR amplification, sequencing	60	30	211	606728	606938
4	**TGGGGAATGATTTGGTAGTTTC**	**GATGCTATCCTTCCGACCATAC**	PCR amplification, sequencing	60	30	4,872	690253	695124
	AAGCACCGGCTAACTCCGTGCC	AGCTGCCCGTGCAGTATCATCG	Sequencing					
	AGCGCAACCCTTATCATCAG	ATGAGCCGACATCGAGGTGC	Sequencing					
	AGGAGGTCATCGGTTCGAAC	AGTCGCTACCGCCATTTCTC	Sequencing					
		CATCGTTTTCCACTTAGCATGG	Sequencing					
		AGGGTGAATCAAGGTTTCGC	Sequencing					
5	**CCACGATCCCCTTGATATAGTT**	**CGGTAACCGTAACTGACAATCC**	PCR amplification, sequencing	60	30	1,846	776037	777882
	TGTCGCGGATGAAGGCATTGAG	ACGTCCTCATTGCCCGGTTCAC	Sequencing					
6	**TTCCCCAGAACTTGATCTGATT**	**CAGATTGTATTGGCAGGAACAA**	PCR amplification, sequencing	60	30	6,166	812018	818183
	GATCACCTTCACGAGCCTTGAG	ACCCACCTGGAGGATATGTACG	Sequencing					
	TGGCGATCGTCTACATGCTG	ACAACTGGCAGCGGATTATC	Sequencing					
	GAACAAATGGCCACGGCCTC	TGATTCCGCCCTGGTAGAG	Sequencing					
	ACGTCACCAACCAAATCAGC	CCATAACTAAACCGGCCATG	Sequencing					
7	**CATATAGTCGAGCGTCTGTTCG**	**GGTTCTGCTCCAGGACTTTACC**	PCR amplification, sequencing	60	1	302	913748	914049
8	**ATACCGTGGTGCTCGTAGAAAT**	**GAATACTCCAGCAGCAGATCCT**	PCR amplification, sequencing	57	25	44	985548	985591
9	**GAAGCTACACCATCAGCGAGTA**	**GTAATATCAACGGTCGACACCA**	PCR amplification, sequencing	60	1	234	1007442	1007675
10	**ACGGCATCTGTTTTCAAATCTT**	**GGAACCTGTTTCGTACCTTTTG**	PCR amplification, sequencing	60	30	3,292	1045471	1048762
	GCCATAGATGACCTGTTCTTTG	TCCAGCTCGACTGCAAGCCTTG	Sequencing					
	TACACGGCTATAGAACGACG	ACGTTCTGACAGTGCGTTCG	Sequencing					
	TATCCTGTTCCGCTGGTTCC	GTGACTTGAAGCTCTTGATG	Sequencing					
11	**CAAGATAGGGATTCATTCTCACG**	**AAAGAGATCCATGGTCAGGGTA**	PCR amplification, sequencing	60	1	305	1070868	1071172
12	**TTGGTAGTTTCGGTGTTCCTTT**	**CACATATCTCATCGACTGAGAC**	PCR amplification, sequencing	58	30	4,872	1235830	1240701
	GAGAGGATGATCAGCCACAC	CTGCCCGTGCAGTATCATCG	Sequencing					
	AGCGCAACCCTTATCATCAG	AGTCGCTACCGCCATTTCTC	Sequencing					
	AGGAGGTCATCGGTTCGAAC	CATCGTTTTCCACTTAGCATGG	Sequencing					
		AGGGTGAATCAAGGTTTCGC	Sequencing					
13	**TTTCCTGGAGGACTATGCATTT**	**GGGCTATCTGCTTCTTTTCAGA**	PCR amplification, sequencing	60	1	157	1288215	1288371
14	**CTGGCGAATGTTTTCCATCTAT**	**TGAAAGATATTGAGCGGGAGAT**	PCR amplification, sequencing	60	1	279	1305843	1306121
15	**TTTCTGGCTCATCCTCAATCAT**	**ATCAAGGTCAAGATCCTGGAAA**	PCR amplification, sequencing	60	1	369	1386224	1386592
16	**CCGTAGTGAAGGGAGAGTATGC**	**GGGATATAGGTCCAGATGGTCA**	PCR amplification, sequencing	60	1	285	1606542	1606826
17	**GCTGATGAGACCTGTAGCACTG**	**AAAGGAAGCGCAAGTTTTACCT**	PCR amplification, sequencing	65	40	1,078	2268239	2269316
	AGGCTGCTGAGGCGCCATAG	AAAGGCCGTTGCCGCCAGCACGAT	Sequencing					
	TTGATGCCGAGGCCAGGCGGGG		Sequencing					
18	**CCGGAGTCGATAGTACATGAGA**	**GCAACGAGTGGTGGTACTTCAC**	PCR amplification, sequencing	60	1	315	2312066	2312380
19	**CTTGACGAAGAACGACATGAAC**	**CTTACGTTCGCCGTCTGGTACT**	PCR amplification, sequencing	60	30	5,412	2590955	2596366
	AATACCGATTCACCCACCGATG	GATAATCACCTGCATGATCACC	Sequencing					
	CAGCTCGTAGTAGATCTTGC	CAGTACGAGATCACCCGCTG	Sequencing					
	AACTCGGAGAGCATGCTGTC	CGGAGCCATCAAGGTCTGTC	Sequencing					
	ATCTTCGCCGTGGACGTGGAAC	CTCTCTCATCTATCGCTACC	Sequencing					
		TCGGCGAGGAAACGCTTCTCTC	Sequencing					
20	**GGACCACTTAACACAACCCTGT**	**CAACTATCCTGCGTGAATTGAG**	PCR amplification, sequencing	60	30	5,723	2663241	2668963
	CACAGGTGTTAAGGAACAGATG	GATCACGCTGGTGAACAGGCTG	Sequencing					
	ATCTCGTCCAGGAAGATCGTG	ACAGGGACGATCACCATGAG	Sequencing					
	TGAGGATGGCATCGTCCACG	GCCGACTGGCATGGATATTG	Sequencing					
	CCTTGATATCCAGGCGCTTG	ATAACCTCCTGGGCGATCTC	Sequencing					
21	**CCCAAATATACCGCACTATTCC**	**TTCAATGGTGAAATCGATGTGT**	PCR amplification, sequencing	60	30	1,895	2686577	2688471
	CTCGACTCTGGCGGAGATGAAC	ATACTCACCGAGGACACCAATG	Sequencing					
22	**CTTGATTCCCACTCTCAGGTTC**	**AACGTGTTTCCCTTGGTCATTC**	PCR amplification, sequencing	60	1	248	2742900	2743147
23	**AAATACTCCTGACTGGCCAAAA**	**TTATGAAACGTCGTGAAAATGC**	PCR amplification, sequencing	60	30	7,459	2756485	2763943
	AAGCGGATGCGCTCATTAAC	GTGCCATCAACACTGAAGTG	Sequencing					
	CAGATCGACATCTGTTGACG	GAAATCGAAAGACGAGCCGC	Sequencing					
	AACATCACCTGTCCGCTTTC	GACAAACACACCACACAAGG	Sequencing					
	CTTCTTCCAGGGTGAAGATC	AGGTTGTCCTGCGGTTGATG	Sequencing					
	CTTTCGAGTTGCAGCATCAG	AAGAGCATCCGGGAGATCTC	Sequencing					
	GTGCTGGATGTCGCTCATAG	GGGACACGGCAAAGCATTTC	Sequencing					
24	**CGGTAAATCCTGAATTCCATCC**	**ATTCCCTCGACTTCAATCAATC**	PCR amplification, sequencing	60	1	210	2777910	2778119
25	**CTCCATCATGTGTTCAGGGTTA**	**GGTTTCTGGTAGTGTTGGAAGC**	PCR amplification, sequencing	60	1	843	1874360	1875202
26	**ATCCACCTCGTAGTCATGAAAG**	**CCAGATTCTCTACTCGGTGGTG**	PCR amplification, sequencing	60	30	3,285	2898646	2901930
	TCTTCTGGCCGATCTGACGGAG	ATAACGTGGAGACCCTTGCGAC	Sequencing					
	TGTAGCCGTCCACCGGAATC	GCTACTTCCGGAATGAATACG	Sequencing					
27	**TCGTAAATATCGATTCGTGGTG**	**GTGACGGAGATAACGCTAAACC**	PCR amplification, sequencing	60	30	1,024	2949114	2950137
	GGCTTTGACGACAAGATCATC		Sequencing					
	GTCAGTCATGGCGGAACAAC		Sequencing					
28	**TCGTAAATATCGATTCGTGGTG**	**GTGACGGAGATAACGCTAAACC**	PCR amplification, sequencing	60	30	17	2954455	2954471
29	**GAACCGAAGACCTTCATCCATC**	**GAAATCGTCGCAAAGGTTAAAG**	PCR amplification, sequencing	60	1	893	3026979	3027871
30	**CCCGAACTATTCGATAATGGAG**	**GTCACTACTCACCGTGGTTGCT**	PCR amplification, sequencing	60	30	3,188	3043295	3046482
	GCTCGTCCTTGTCGCCATTGAG	GAACATGTACGGCGTGCACTGC	Sequencing					
	TTACCAAGGAAGTGCTCTAC	ATCGGGAGAAGAGGTTGCAG	Sequencing					
	CCAATGGAGGCAGAATCAAG	CGAGGGCATCGACATGGATG	Sequencing					
31	**CCCCAGAAACATAGGGTGAATA**	**GTGGTGCCGTTGTAGACATAGA**	PCR amplification, sequencing	60	30	3,554	3427838	3431391
	AGCTCGGTGCCAATGAAATTCG	TGATCTTCAGGGCGTCGTTCAC	Sequencing					
	GCCACTTTGAAGGTTATGAC	GATAGGTGCCGTTATAGAACC	Sequencing					
32	**CCTGAAGATCAATGAGGGAAAG**	**ATTGTACTCGGTCATCCACCTC**	PCR amplification, sequencing	60	1	173	3511982	3512154
33	**AACCACCAGCACATCAGAAG**	**GCGCCAACCACCAGCACATC**	PCR amplification, sequencing	57	25	4,408	3627984	3632391
	GTTGTCCATTGAACAGCTGG	CCAGCTGTTCAATGGACAAC	Sequencing					
	CAAGTAATTCCAAATGGAGCG	GATGATTACCTGCTGCATTGC	Sequencing					
	GCAATGCAGCAGGTAATCATC	GAAAGCTCCTTCCCATCCTTCG	Sequencing					
	CCGTGTTCCTAAATTCTTGAGG	AAGCCGCTACCTGAAGGCTTC	Sequencing					
	CGAAGGATGGGAAGGAGCTTTC		Sequencing					
	CTTCAACTTCAGCCGTTCAC		Sequencing					
34	**CACGTCCTTGGGATATTTGTAA**	**GAAGATTACGACCTCTGGCTGA**	PCR amplification, sequencing	60	1	165	3736120	3736284

a The primer sets in bold were used for both PCR amplification and the sequence in each gap.

b PCR conditions used were as follows: 25 cycles of 98°C for 10 s, annealing temperature indicated in the list for each reaction for 5 s, 68°C for extension time indicated in the list for each reaction, and 68°C for 5 min. For all reactions, KOD One PCR master mix (Toyobo) was used. The amplicons were gel purified by Monarch DNA gel extraction kit (New England BioLabs).

### Data availability.

The complete genome sequence of strain YM35 was deposited under GenBank accession number CP074693. The raw reads were deposited in the Sequence Read Archive under BioProject accession number PRJNA742140.
